# *In Situ* Analysis of mTORC1/C2 and Metabolism-Related Proteins in Pediatric Osteosarcoma

**DOI:** 10.3389/pore.2022.1610231

**Published:** 2022-03-22

**Authors:** Anna Mohás, Ildikó Krencz, Zsófia Váradi, Gabriella Arató, Luca Felkai, Dorottya Judit Kiss, Dorottya Moldvai, Anna Sebestyén, Monika Csóka

**Affiliations:** ^1^ Second Department of Pediatrics, Semmelweis University, Budapest, Hungary; ^2^ First Department of Pathology and Experimental Cancer Research, Semmelweis University, Budapest, Hungary

**Keywords:** osteosarcoma, mTOR, pathways, metabolic, metabolic adaptation, pediatric

## Abstract

Activation of the mTOR pathway has been observed in osteosarcoma, however the inhibition of mammalian target of rapamycin (mTOR) complex 1 has had limited results in osteosarcoma treatment. Certain metabolic pathways can be altered by mTOR activation, which can affect survival. Our aim was to characterize the mTOR profile and certain metabolic alterations in pediatric osteosarcoma to determine the interactions between the mTOR pathway and metabolic pathways. We performed immunohistochemistry on 28 samples to analyze the expression of mTOR complexes such as phospho-mTOR (pmTOR), phosphorylated ribosomal S6 (pS6), and rapamycin-insensitive companion of mTOR (rictor). To characterize metabolic pathway markers, we investigated the expression of phosphofructokinase (PFK), lactate dehydrogenase-A (LDHA), β-F1-ATPase (ATPB), glucose-6-phosphate dehydrogenase (G6PDH), glutaminase (GLS), fatty acid synthetase (FASN), and carnitin-O-palmitoyltransferase-1 (CPT1A). In total, 61% of the cases showed low mTOR activity, but higher pmTOR expression was associated with poor histological response to chemotherapy and osteoblastic subtype. Rictor expression was higher in metastatic disease and older age at the time of diagnosis. Our findings suggest the importance of the Warburg-effect, pentose-phosphate pathway, glutamine demand, and fatty-acid beta oxidation in osteosarcoma cells. mTOR activation is linked to several metabolic pathways. We suggest performing a detailed investigation of the mTOR profile before considering mTORC1 inhibitor therapy. Our findings highlight that targeting certain metabolic pathways could be an alternative therapeutic approach.

## Introduction

Osteosarcoma is the most common pediatric bone tumor, accounting for approximately 3% of all malignancies in children (1). Multi-agent chemotherapy and surgical resection of the tumor and the metastases are the cornerstones of the current treatment guidelines. With this multidisciplinary approach, up to 70% of the patients with localized disease can be long term survivors. The prognosis is poorer for patients with primary metastatic, nonresectable, or relapsed disease, and the overall survival in these cases was reported to be around 30%. Metastases at the time of the diagnosis, axial location, and chondroblastic histological subtype can indicate worse prognosis. Despite the advances in chemotherapy and surgical methods, the overall survival rates have not improved in the past few years (2). In order to develop new targeted therapies, better understanding of the molecular mechanisms of osteosarcoma is needed.

Research of cancer metabolism is an area that has gained attention in recent decades. In order to maintain the high proliferation rate, malignant cells need to rewire their metabolic pathways to gain more energy for growth and invasion. These changes consist of upregulated glycolysis (even in the presence of oxygen) also known as the “Warburg-effect,” increased glutaminolysis and lactate synthesis, alterations in lipid metabolism, and tricarboxylic acid cycle/oxidative phosphorylation. As a result of these alterations, several alternative metabolic pathways can be activated in tumor cells (3).

The role of phosphoinositide-3-kinase (PI3K)/protein-kinase B (AKT)/mammalian target of rapamycin (mTOR) pathway in tumorigenesis has been investigated thoroughly in the past years. The serin/threonine kinase mTOR is a master regulator of cell growth and proliferation, it promotes anabolic processes (protein, nucleotide, fatty acid synthesis) and inhibits catabolism. mTOR kinase is found in two different complexes, mTOR complex 1 (mTORC1) and mTOR complex 2 (mTORC2). Each complex contains different proteins as they have distinctive roles and they also differ in inhibitor sensitivity. mTORC1 contains mTOR, regulatory‐associated protein of mTOR (raptor), and G protein β subunit‐like (Gβ L, also known as mLST8), and is sensitive to rapamycin, whereas mTORC2 consists of mTOR, Gβ L, and rapamycin-insensitive companion of mammalian target of rapamycin (rictor), and could be insensitive or less sensitive to rapamycin. The activation of mTORC1 leads to increased protein synthesis, cell growth and proliferation, whereas mTORC2 regulates cytoskeleton reorganization and cell survival (4, 5). Due to mutations of the regulatory proteins/enzymes of the cellular signaling network, constitutive activation of this pathway was described in a variety of tumors. Rapamycin analogues (rapalogs) have been investigated as therapeutic agents in several human malignancies, and successfully used in certain diseases such as renal cell carcinoma and mantle cell lymphoma (6).

The mTOR pathway is critical in tumor survival and progression, and it is potentially activated in osteosarcoma, as it has been already reported, although the mechanisms of this activation are poorly understood. Some upstream (PI3K) and downstream (S6K1, 4EBP1, and eIF4E) effectors of the pathway are overexpressed in osteosarcoma, leading to alterations of cellular transformation and poorer clinical prognosis (7, 8). Inhibition of mTORC1/2 or knockdown of mTOR or rictor prevents proliferation in osteosarcoma cell lines, and the combination of mTOR kinase inhibitor PP242 with cisplatin leads to synergistic antitumor activity (9). Combination with cisplatin or bevacizumab can enhance the effectivity of temsirolimus in osteosarcoma xenografts (10), and rapamycin with spautin-1 (specific and potent autophagy inhibitor-1) was found to be effective in apoptosis induction, and can be a possible treatment option (11). Despite encouraging preclinical data, mTOR inhibitor monotherapies and combined therapies have shown poor antitumor effect in the treatment of osteosarcoma (12, 13).

Better understanding of the different tumor types’ metabolic adaptation changes is crucial in order to improve personalized therapies. Our aim was to characterize the mTOR profile and certain metabolic alterations in pediatric osteosarcoma to determine the interactions between the mTOR pathway and metabolic pathways.

## Materials and Methods

### Materials

We collected tissue samples of 28 patients diagnosed with osteosarcoma between 2009 and 2016 in the First Department of Pathology and Experimental Cancer Research, Semmelweis University (Budapest, Hungary). The tumors were obtained by open biopsies at the Semmelweis University Department of Orthopedics, the patients were treated in Semmelweis University’s Second Department of Pediatrics. The study was approved by the Ethics and Scientific Committee of Semmelweis University (project identification code: 99/2018).

All of the samples were taken from the primary tumor, no metastatic or relapse lesions were investigated. Clinicopathological data, such as age, sex, and histological subtype were collected from medical records (Table 1)*.*

**TABLE 1:** Clinicopathological characteristics of the studied patients.

Histology subtype	Patients (N = 28)
Osteoblastic	22
Other	6
	Chondroblastic: 3
	Fibroblastic: 2
	MFH-like: 1
Gender	No.
Male	18
Female	10
Age at diagnosis	Years
Median (range)	12.9 (2.8–17.9)
Metastasis at the time of diagnosis	No.
No metastasis	14
Pulmonary metastasis	14
Chemotherapeutic response	No.
Good	22
Poor	5
Overall survival at 5 years	64.20%

We have no information of one patient’s chemotherapy response as no postoperative sample was available.

Tissue microarrays (TMAs) (6 × 9; diameter, 2 mm) with double cores per patient were prepared from the formalin-fixed, paraffin-embedded tissue samples (TMA Master; 3DHistech, Budapest, Hungary). Representative areas of formalin-fixed paraffin-embedded blocks were selected based on hematoxylin-eosin stainings by an experienced pathologist. Testis, liver, and tonsil samples were used as controls.

### Patient Selection, Treatment and Clinical Data

Cases were selected from osteosarcoma patients who were treated in the Semmelweis University’s Second Department of Pediatrics between 2009 and 2016. All patients from this period with available biopsy samples were included in this study.

Therapy included complete surgical removal of all detectable tumors as well as multiagent neoadjuvant and adjuvant chemotherapy. The chemotherapy regimen included several or all of the following drugs: doxorubicin, high-dose methotrexate with leukovorin-rescue, cisplatin, and ifosfamide. Resection was either limb-sparing surgery or amputation. Chemotherapeutic response was evaluated after the surgery by determining the level of necrosis of the resected tumor. If ≥ 90% of the tumor showed necrosis, it was determined as “good response,” less than 90% necrosis of the tumor was considered as “poor response” (14).

Pulmonary metastases were also surgically removed. Patients were treated in an identical manner, irrespective of histological subtype. Radiotherapy was given as a palliative treatment in one case, otherwise it was not part of the first-line therapy. After the treatment, patients had regular follow-ups. Each visit includes a history and physical examination, chest CT and bone scintigraphy is performed regularly in order to detect local recurrence or metastases. Follow up period was 5 years in this study.

### Immunohistochemistry

The immunohistochemistry was performed on 4 μm thick sections of the TMA, and it was previously validated in our laboratory. After the deparaffinization and endogenous peroxidase blocking, we performed antigen retrieval for 20–30 min (citrate buffer pH 6). Slides were incubated in primary and secondary antibodies, followed by DAB (Dako, Carpinteria, CA, United States) chromogen and hematoxylin counterstaining. The primary antibodies are summarized in Table 2.

**TABLE 2:** Antibodies and their conditions used in this investigation.

Antibody	Antigen	Clone	Dilution	Secondary antibody	Phosphorylation site	Significance	Supplier
mTOR markers	pmTOR	#2976	1:100	Novolink	Ser2448	mTORC1, mTORC2	Cell Signaling (Boston, MA, United States)
	pS6	#2211	1:100	Novolink	Ser235/236	mTORC1	Cell Signaling (Boston, MA, United States)
	rictor	A500-002A	1:1000	Vectastain		mTORC2	Bethyl Laboratories, Inc. (Montgomery, TX, United States)
Metabolic markers	PFK	#8164	1:100	Novolink		Glycolysis	Cell Signaling (Boston, MA, United States)
	LDHA	#3582	1:400	Novolink		Glycolysis	Cell Signaling (Boston, MA, United States)
	ATPB	ab14730	1:100	Novolink		Oxydative phosphorylation	Abcam, Cambridge, United Kingdom
	G6PDH	ab133525	1:100	Novolink		Pentose phosphate pathway	Abcam, Cambridge, United Kingdom
	GLS	ab156876	1:200	Novolink		Glutaminolysis	Abcam, Cambridge, United Kingdom
	CPT1A	ab128568	1:500	Novolink		Beta oxydation of fatty acids	Abcam, Cambridge, United Kingdom
	FASN	#3180	1:50	Novolink		Long chain fatty acid synthesis	Cell Signaling (Boston, MA, United States)

To characterize mTOR complex activity *in situ*, we used anti-pmTOR, anti-phosphorylated ribosomal S6 (pS6), and anti-rictor antibodies. Rictor and pmTOR results together were interpreted to confirm mTORC2 activity, mTORC1 activity was evaluated by the expression of pS6 and pmTOR.

To examine the metabolic activity features, we applied anti-LDHA (lactate-dehydrogenase A), anti- PFK (phosphofructokinase), anti- G6PDH (glucose-6-phosphate dehydrogenase), anti-ATPB (βF1-ATPase), anti-GLS (glutaminase), anti-CTP1A (carnitine O- palmitoyltransferase 1), and anti-FASN (fatty acid synthase) antibodies (Table 2).

To assess the immunohistochemistry stainings, we calculated the histoscore (H-score). With this semiquantitative analysis we scored the staining in a scale from 0 to 300. The H-score was calculated by multiplying the staining intensity (0,1+,2+,3+) by the percentage of positive cells—as previously described in other studies (15, 16). Samples with an H-score of 100 or higher were considered “high”, scores less than 100 were considered “low”. H-scores were evaluated by two independent investigators. We performed statistical data analysis according to histological subtype, age at diagnosis, metastasis at diagnosis and survival.

### Statistical Analysis

Statistical analysis was performed using IBM SPSS Statistics Program version 27.0.1. (SPSS Inc., Chicago, IL, United States). We used two-sample t-test, one-way Anova test, Mann Whitney U-test, Fisher’s exact test, and Spearman’s rank correlation. A p value less than 0.05 was considered statistically significant. We compared our results according to metastases, age at diagnosis, histological subtype, response to chemotherapy, relapse, and survival.

## Results

### Expression of mTOR-Related Proteins

To characterize mTOR activity, we chose to use pmTOR antibody, which detects the active form of mTOR kinase. In order to analyze mTORC1 and mTORC2 activity, we used two more immunostainings, pS6, and rictor. The amount of pS6 correlates with mTORC1 activity, and rictor is a reliable marker for the quantity of mTORC2. Evaluation of the pmTOR, pS6, and rictor activity together can give us a result regarding the specimen’s mTOR activity. Co-expression of pmTOR and pS6 was considered as the activation of the mTORC1 complex. Assessment of mTORC2 activity was based on the co-expression of pmTOR and rictor. Rictor immunopositivity without pmTOR or pS6 positivity was considered as the potential activation of the mTORC2 complex. The positivity of pmTOR without pS6 or rictor was determined as potential mTOR activity.

**FIGURE 1:**
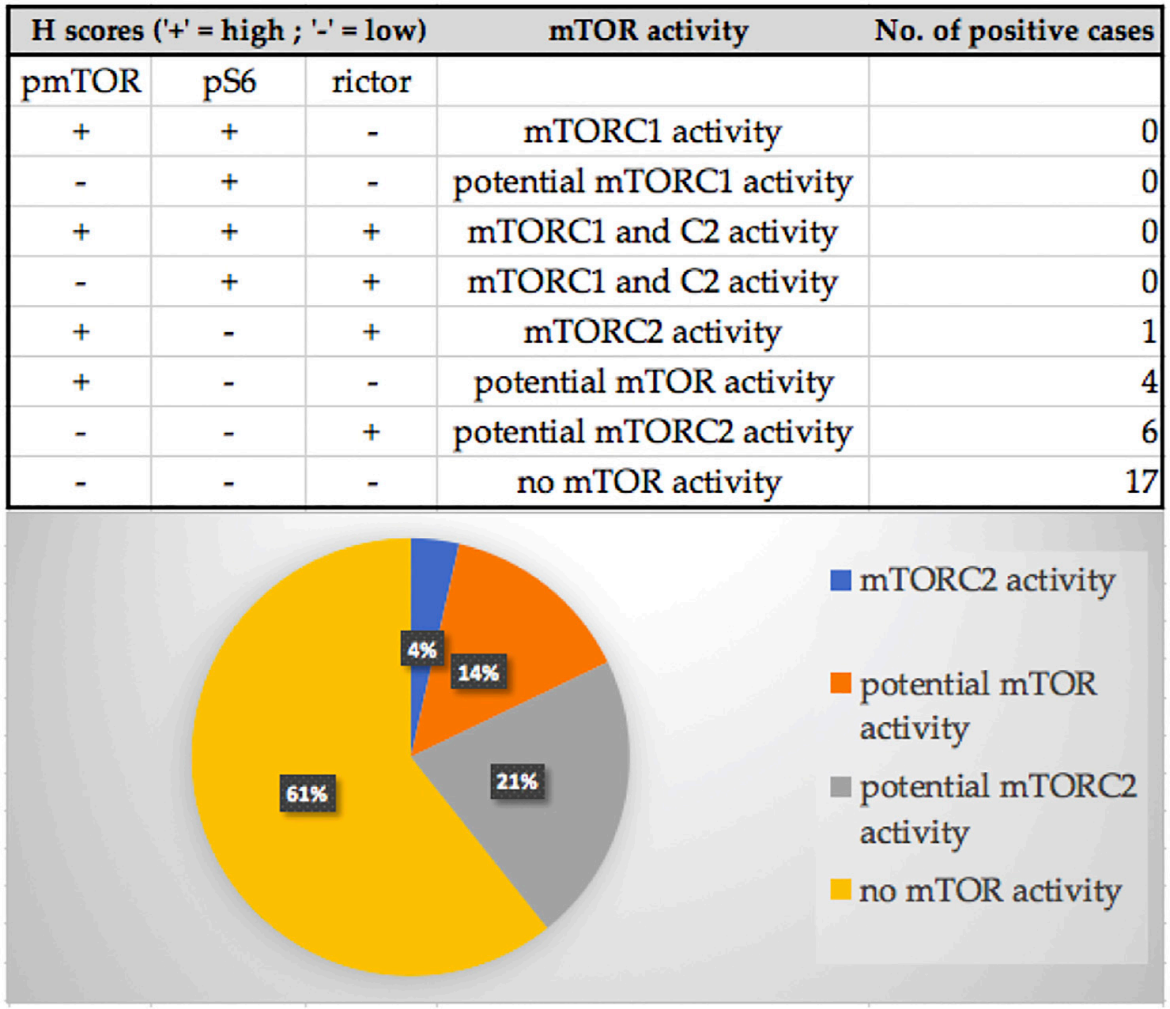
The distribution of the pmTOR, pS6 and rictor immunostaining results and mTOR activity characterization of the samples. Most of the cases (61%) showed low mTOR activity, although 21% of the samples showed potential mTORC2 and 14% showed potential mTOR activity. We found mTORC2 activity in only one sample (4%).

**FIGURE 2:**
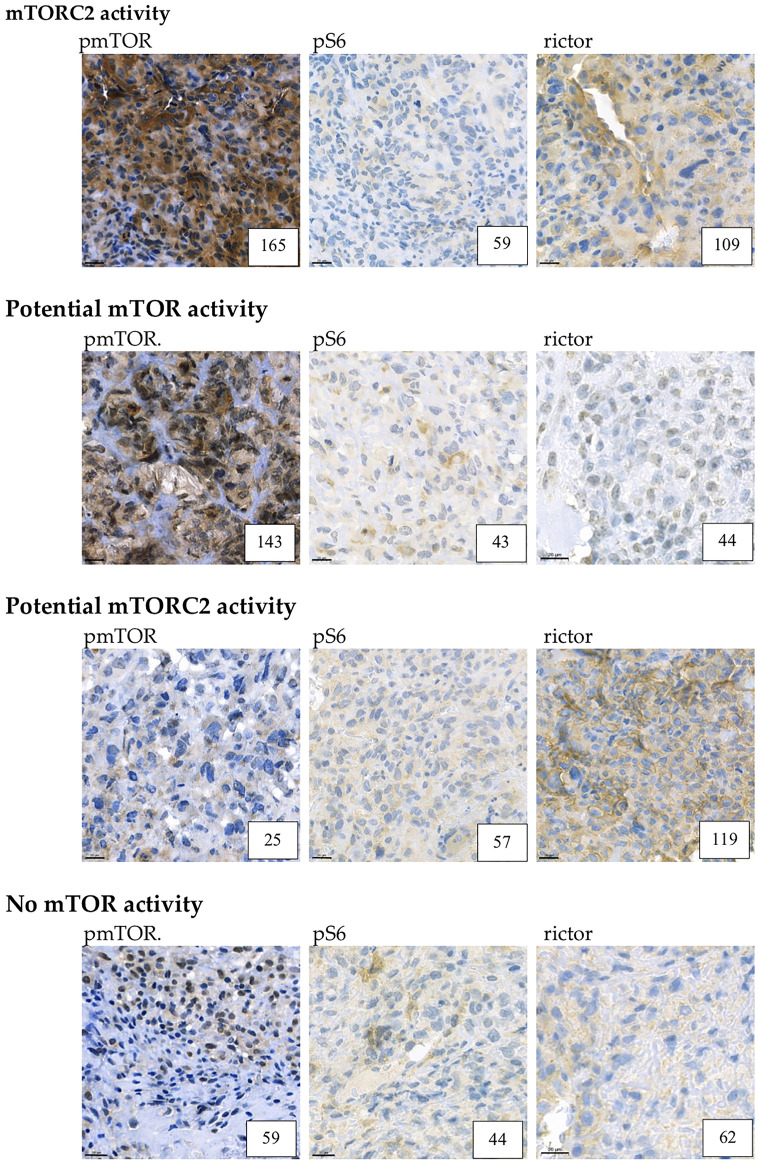
Example for mTORC2, potential mTOR, potential mTORC2 and low mTOR activity. Representative pmTOR, pS6 and rictor immunohistochemical stainings are presented. The numbers in the right lower corner are the given H-scores for each sample. Pictures were taken with CaseViewer 2.3. Software (3D Histech Ltd. Budapest, Hungary). The scale bar shows 20 μm.

In the evaluated osteosarcoma samples, we observed low pS6, rictor, and pmTOR activity. Most of the cases (61%) showed low mTOR activity, although 21% of the samples showed potential mTORC2 and 14% showed potential mTOR activity. We found mTORC2 activity in only one sample (4%). Figures 1, 2 *.*

### Expression of Metabolic Pathway-Related Proteins

#### Glycolysis, Oxidative Phosphorylation, and Footprints of Warburg-effect in Osteosarcoma Cells

**FIGURE 3:**
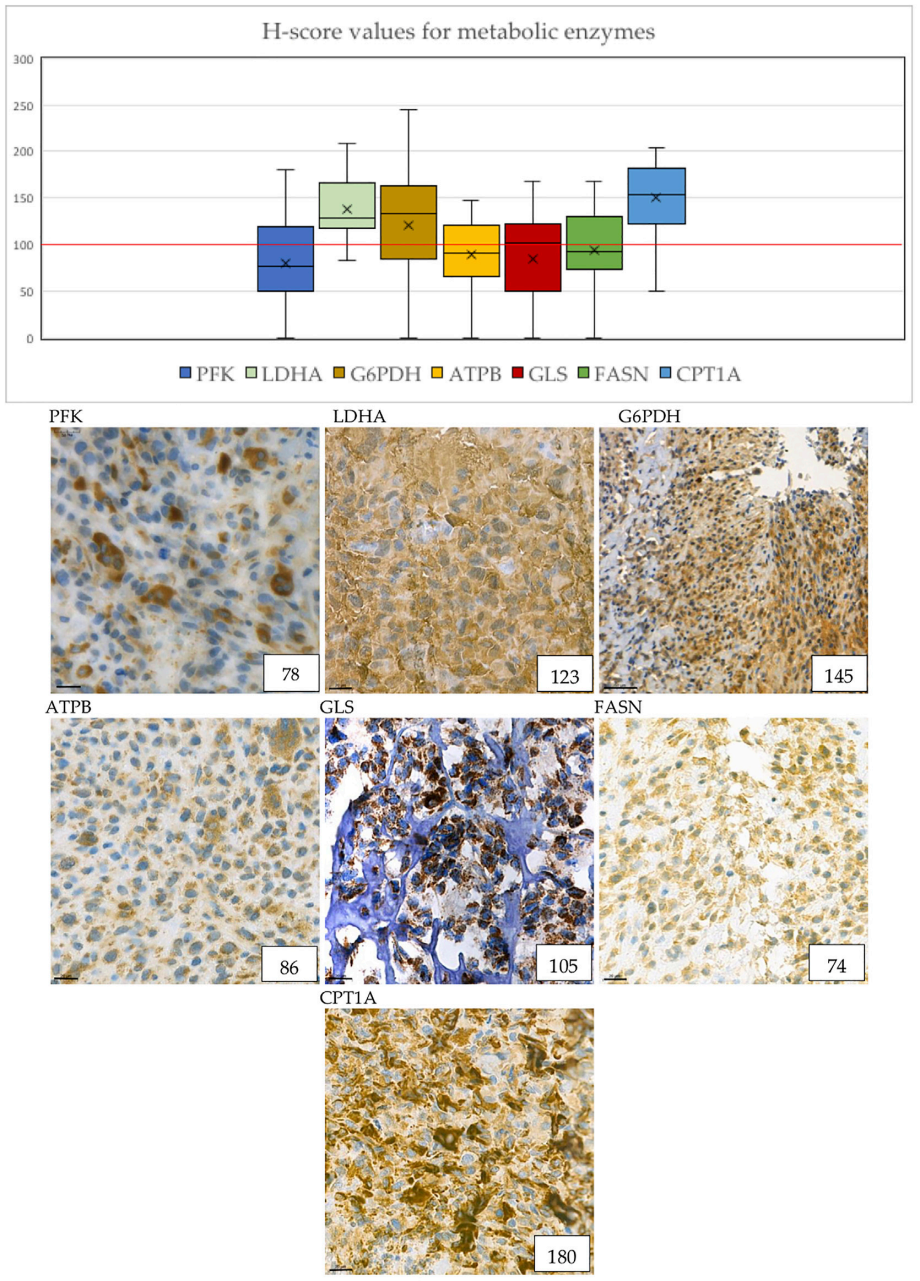
Metabolic marker expression in osteosarcoma cases. The distribution of H-scores are visible on the boxplot graph. “X” signs the mean, horizontal lines sign the median values of H-scores. The numbers in the right lower corner are the given H-scores for each sample. Evaluation of metabolic markers showed that LDHA, G6PDH, GLS and CPT1A expression was high in most of the cases. ATPB and GLS shows granular pattern due to mitochondrial localization. Pictures were taken with CaseViewer 2.3. Software (3D Histech Ltd. Budapest, Hungary). The scale bar shows 20 μm.

Cytoplasmic immunoreactivity of PFK was low in most of the samples, only 32% of the cases showed high H-scores, the median of the H-score values was 77. In contrast, the expression of LDHA—the marker of anaerobic glycolysis (Warburg-effect)—was high in 93% of the cases. We observed low ATPB expression (only 39.3% of the cases were evaluated as high), which suggests that oxidative phosphorylation is less pronounced in the examined samples. These data underline the importance of Warburg-effect in osteosarcoma cells (Figure 3).

#### Importance of the Pentose Phosphate Pathway

We evaluated the expression of G6PDH as a marker of the pentose phosphate pathway. 71.4% of the cases showed high expression of G6PDH, which suggests that this pathway may have a crucial role in the metabolism of osteosarcoma cells. In addition, the H-score values of G6PDH turned out to be the second highest of all stainings with the median value of 133.5 (Figure 3)*.*

#### Glutaminolysis

Glutaminase (marker of glutaminolysis) was highly expressed in 53.6% of the cases, with a median H-score of 102, suggesting that glutamine may be used as an alternative bioenergetic substrate in osteosarcoma tumorigenesis (Figure 3)*.*

#### Fatty Acid Beta Oxidation and Long Chain Fatty Acid Synthesis

CPT1A is a rate-limiting enzyme in fatty acid beta oxidation. The expression of this enzyme was positive in 89.3% of the cases, and the median of the H-scores showed the highest results of all immunostainings. This data underlines the importance of fatty acid beta oxidation in osteosarcoma cells.

FASN is an enzyme involved in endogenous lipogenesis, its function is to catalyze the synthesis of long-chain fatty acids. We found that FASN was highly expressed in 42.9% of the cases (Figure 3)*.*

### Association Between Protein Expression and Clinicopathologic Parameters

**FIGURE 4:**
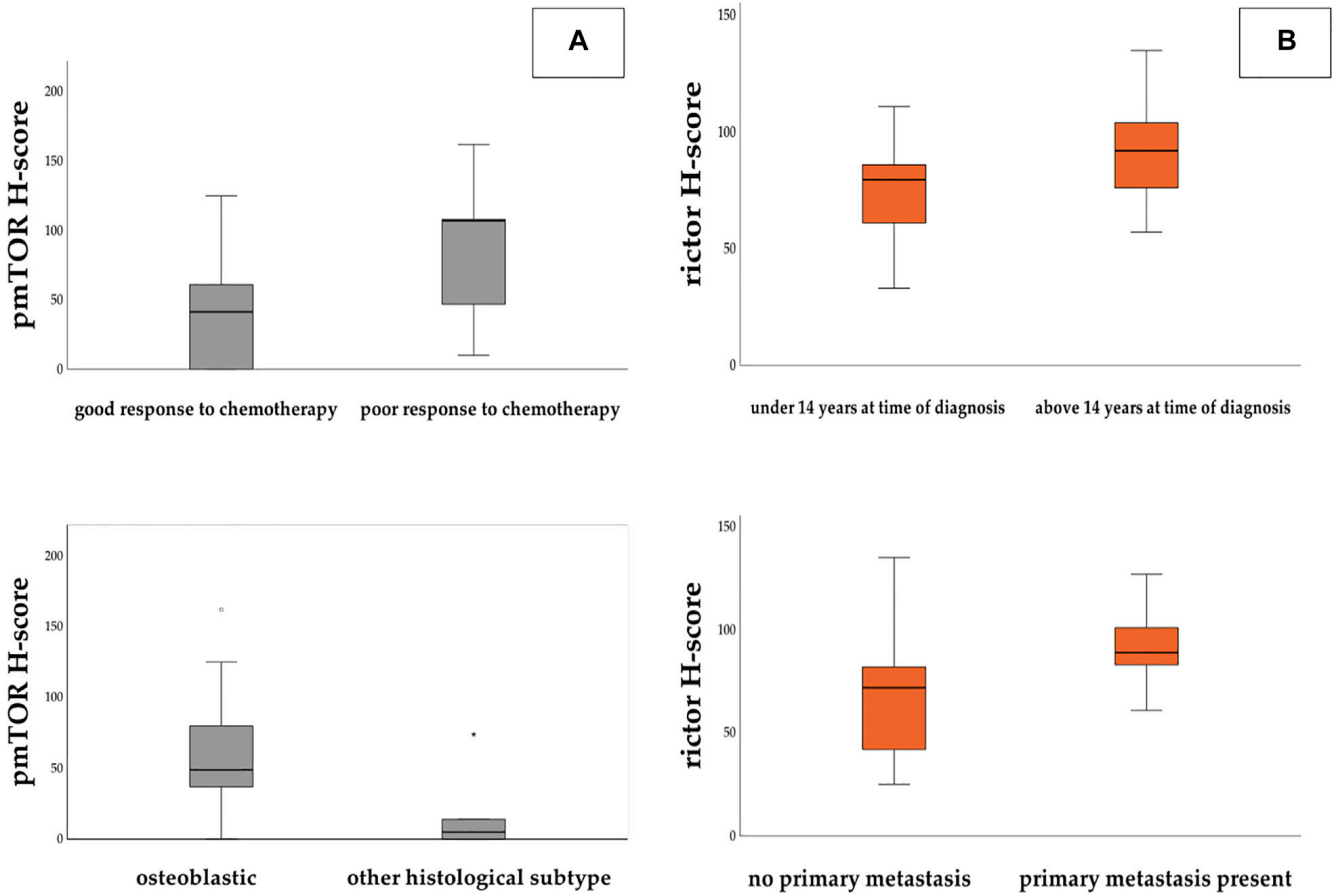
Panel **(A)**: H-score values for pmTOR were found significantly higher in those patients who showed poor response to chemotherapy. H-score levels were significantly lower in non-osteoblastic subtype. Panel **(B)**: H-score values for rictor were significantly higher in those patients who had metastasis at the time of the diagnosis and who were older than 14 years.

Based on the expression of pmTOR, mTOR activity was found to be low in most of the cases. However, we found that pmTOR expression was significantly lower in patients with good histological response to chemotherapy (Fisher’s exact test, *p* = 0.031). Expression of pmTOR was also significantly lower in non-osteoblastic histology subtype (Mann-Whitney-U test, *p* = 0.029) (Figure 4A). Regarding rictor expression, our results showed that H-scores for rictor were significantly higher in metastatic disease (two-sample t-test, *p* = 0.019), and lower in those patients, who were diagnosed before the age of 14 (Fisher’s exact test, *p* = 0.038) (Figure 4B).

**FIGURE 5:**
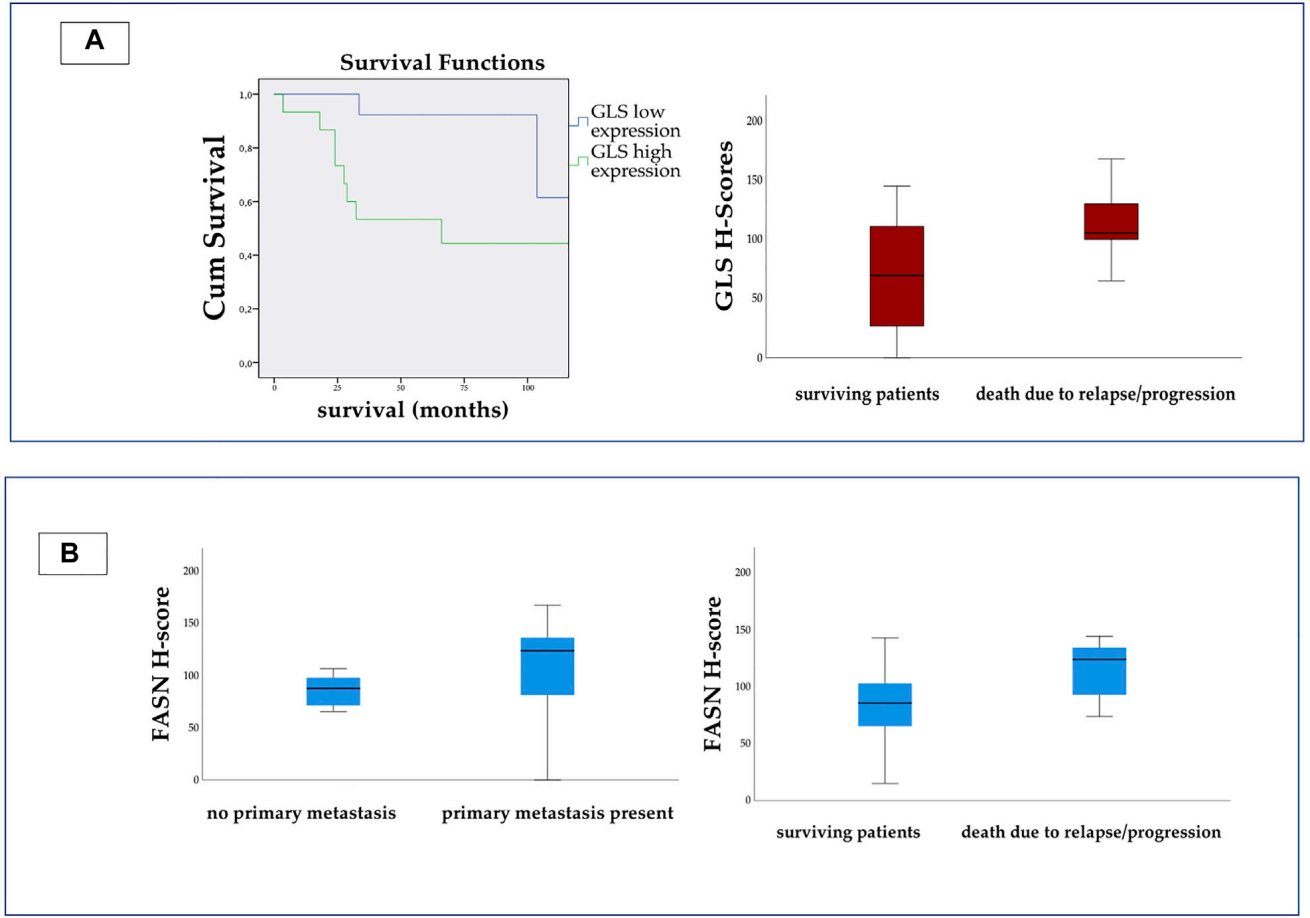
On the left side of Panel **(A)** Kaplan-Meier curve shows significant difference between the survival of patients with high and low GLS expression. (Log Rank *p* = 0.019.) On the right side we present the H-score values for GLS. The scores were significantly higher in patients, who died due to relapse or progression. On Panel **(B)** we present the H-score values for FASN. The expression was significantly higher in metastatic disease, and in patients who died due to relapse or progression.

We found statistically significant elevation of GLS expression in those patients, who died due to progression or relapse (Fisher’s exact test, *p* = 0.043 and Mann-Whitney-U test, *p* = 0.02) (Figure 5A).

The H-score for FASN appeared to be significantly higher in patients with metastatic disease (Fisher’s exact test, *p* = 0.027) and also in those who died due to progression or relapse (Fisher’s exact test, *p* = 0.039 and Mann-Whitney-U test, *p* = 0.036) (Figure 5B).

### Correlation Between mTOR and Metabolic Pathway-Related Proteins

**FIGURE 6:**
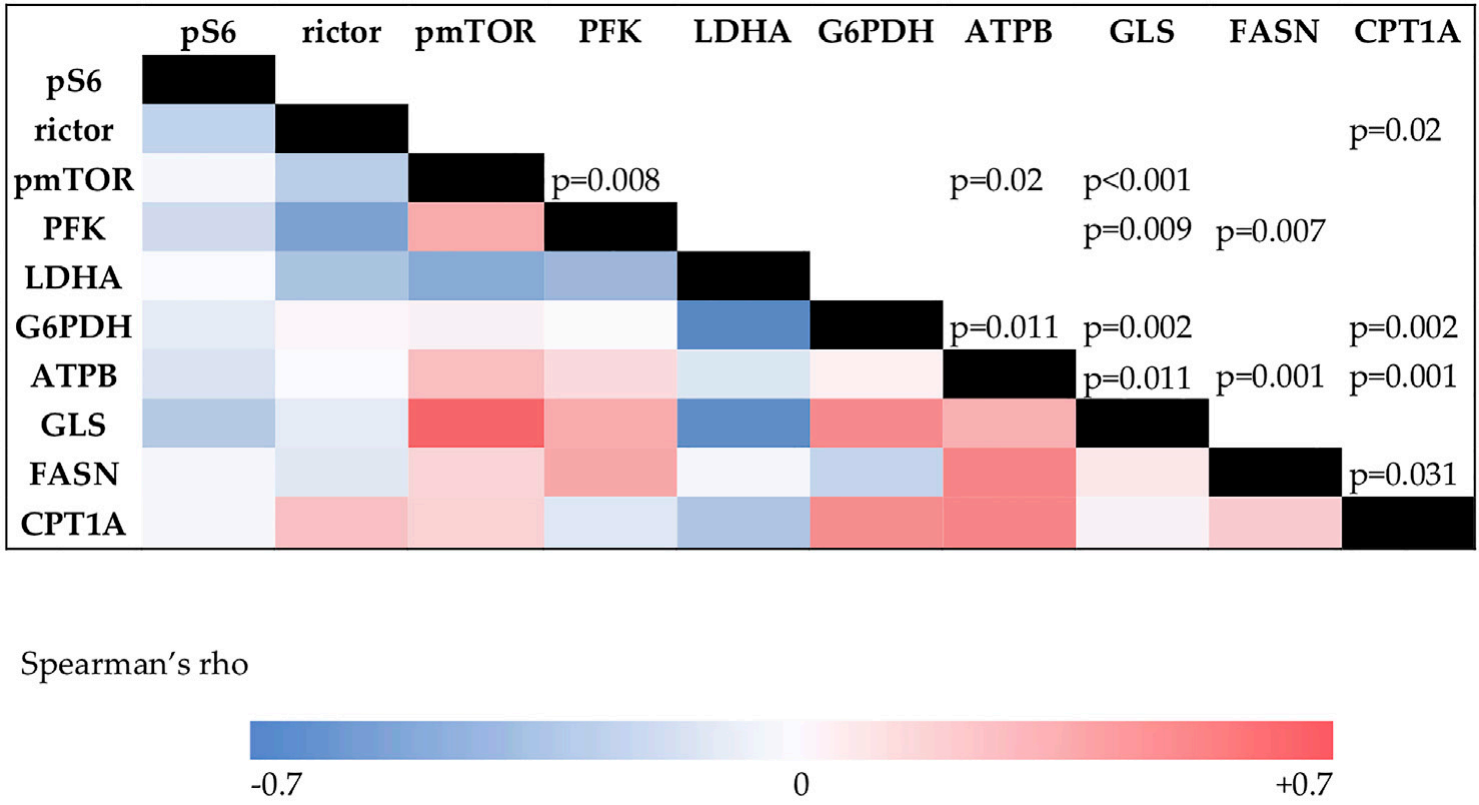
The results of the Spearman’s correlation analysis. The color intensity changes according to the Spearman’s rho value. Correlation coefficient above 0.4 is considered positive, under −0.4 is considered negative correlation. p values for significance are visible in the upper triangle.

Data analyses with Spearman correlation test showed multiple associations between mTOR activity and enzyme expression in this study. We found positive correlations between pmTOR and PFK (R = 0.489 *p* = 0.008), pmTOR and ATPB (R = 0.439 *p* = 0.02), and also between pmTOR and GLS expression (R = 0.647 *p* < 0.001). Rictor and CPT1A showed positive correlation as well. (R = 0.431 *p* = 0.02) (Figure 6)*.*

## Discussion

Osteosarcoma is an aggressive bone sarcoma, affecting mainly children and adolescents. In the 1960s the treatment of this disease was surgery alone, resulting in poor survival rates of 11%. The initiation of adjuvant chemotherapy in the 1970s increased 5-year survival rates up to 70%, but no significant improvements have been made since (2, 17). The survival of relapsed patients, or cases with primary progression remains poor. In order to improve outcome, it is crucial to investigate molecular mechanisms behind osteosarcoma tumorigenesis and progression. Our aim was to characterize the mTOR and metabolic profile of osteosarcoma cases, and to determine potential targets for personalized therapies.

Numerous preclinical data suggest the importance of mTOR activity in osteosarcoma cell proliferation and survival. Investigations with NVP-BEZ235, a PI3K-mTOR dual inhibitor suppressed osteosarcoma cell growth *in vitro* and *in vivo* (18). Similar results were found with the novel mTORC1/C2 dual inhibitor RES-529, which potently inhibits human osteosarcoma cell growth (19). mTOR inhibitors alone, or in combination have been also explored in phase I and II trials (20). Everolimus, mTORC1 inhibitor, combined with sorafenib induced partial response in 5%, and disease control in 63% of the studied 38 osteosarcoma patients (21). Sirolimus, mTORC1 inhibitor, was combined with gemcitabine in a recent phase II trial, resulting in low response rate (6%), but 42% of the patients had stable disease at 4 months of treatment (22). As the data suggest, inhibition of the mTOR pathway has been expected as a promising therapeutic approach, but mTOR inhibitors have not proved their efficacy in osteosarcoma treatment, only in combination with different cytotoxic agents.

It is proved that the mTOR pathway has a potential role in facilitating metastasis. In a murine model of osteosarcoma, rapamycin reduced tumor cell metastasis by blocking the mTORC1 pathway (23). Even though our results suggested low mTOR activity in most of the samples, we found significantly higher H-scores of rictor in primary metastatic diseases. Similar results were found in metastatic lung adenocarcinoma (24). Consistent with these findings, pmTOR activity turned out to be higher in poor responders. In addition, the only patient with mTORC2 activity had a primary progressive disease, showed no clinical response to chemotherapy, and passed away due to disease progression. We observed low expression of rictor in young patients. Younger age at time of diagnosis is associated with better survival (25, 26). We also found significant difference in pmTOR activity between the osteoblastic subtype and other histological subtype group, but due to the low number of non-osteoblastic cases, this data cannot be considered representative. Our results suggest the importance of the mTOR pathway in disease progression and metastasis progression.

Considering our results, we suggest to perform *in situ* mTOR activity profiling before administering mTOR inhibitor therapy for osteosarcoma patients. The most widespread mTOR pathway inhibitors are the mTORC1 inhibitors of the rapalog family. Our results suggest low mTORC1 activity in osteosarcoma, which could stand behind the failure of rapalog therapy in this malignancy.

The mTOR pathway plays a key role in the altered metabolic processes of malignant cells (27, 28). The metabolic profiles of osteosarcoma cells were investigated by characterizing enzymes that play a role in glycolysis (PFK and LDHA), oxidative phosphorylation (ATPB), pentose-phosphate pathway (G6PDH), glutaminolysis (GLS), fatty acid beta oxidation (FASN) and long chain fatty acid synthesis (CPT1A). Malignant cells generate huge amounts of energy for their survival by anaerobic glycolysis (29). By suppressing this process in osteosarcoma cells *in vivo* with the combination of glycolysis inhibitor 2-deoxyglucose (2DG) and adriamycin, a potent antitumor effect was observed (30). We also detected elevated LDHA levels in most of our samples, and moderate expression of ATPB. These findings suggest the hyperactivity of Warburg effect -related enzymes and the lower importance of oxidative phosphorylation in osteosarcoma tissue *in vivo*. Targeting the glycolytic process has been demonstrated to be effective for controlling growth and enhancing anticancer therapies (31, 32).

Enhanced pentose-phosphate pathway activity of numerous malignancies has also been reported (33, 34). In a recent study, osteosarcoma cell growth inhibition was observed by suppressing the level of G6PDH (35). Elevated G6PDH levels in our samples underline the importance of the pentose-phosphate pathway in the nucleotide biosynthesis of osteosarcoma cells.

The importance of glutaminolysis has also been widely investigated in malignant diseases (36). In metastatic cases, cells rely on glutamine as a nutrient for proliferation *in vitro* and *in vivo*. In a recent study, inhibition of glutamine anaplerosis with GLS-1 inhibitor CB-839 reduced the growth of primary osteosarcoma progression and metastasis progression in mouse (37). Our results also suggest a high glutamine demand in progressive osteosarcoma cases, as the H-score levels were significantly higher in those patients who died due to relapse or progression.

Some tumors are dependent on lipids for their progression and survival (38). The characterization of lipid metabolism was also performed in this study. According to our results, high expression of CPT1A suggests that fatty acid beta oxidation may play an important role in osteosarcoma metabolism. Pharmacological inhibition of CPT1A with etomoxir was mostly used in research, but some experiments also indicated promising anti-tumor results (39). Previous experimental evidence has suggested that fatty acid synthase (FASN) may be involved in cancer metastasis, and have been investigated as a therapeutic target (40, 41). The role of FASN in progression and metastasis development in osteosarcoma was demonstrated in previous studies (42). In our study, the expression of FASN was significantly higher in metastatic cases and in patients who died, than those without metastasis and who survived, showing similar results to previous studies (43). This result suggests that FASN might play a role in metastasis development in osteosarcoma.

We also investigated the correlations between metabolic markers and mTOR activity. We found positive correlation between pmTOR and GLS, PFK, and ATPB, suggesting that activated mTOR enhances the activity of certain metabolic pathways as a master regulator. Similar findings were described in pediatric rhabdomyosarcoma as well (44).

Despite the low number of cases available for our studies, our results emphasize the importance of metabolic alterations of osteosarcoma cells. Further investigation of metabolic plasticity may lead to better understanding of therapy resistance and new ways of targeted therapies. Our results indicate potential targets for therapy, but it is still unknown whether it would result in a clinically significant outcome.

In conclusion—based on our results—we suggest performing detailed mTOR profile characterization before administering mTOR inhibitor therapy for osteosarcoma patients. mTOR activation is linked to several metabolic pathways, such as enhanced glycolysis, oxidative phosphorylation, and glutamine demand. These findings may be an explanation of therapeutic failure in this malignancy. Overexpression of GLS and FASN may be a prognostic factor in survival.

## Data Availability

The raw data supporting the conclusion of this article will be made available by the authors, without undue reservation.
